# Does diabetes mellitus impair the clinical results of total knee arthroplasty under enhanced recovery after surgery?

**DOI:** 10.1186/s13018-023-03982-4

**Published:** 2023-07-10

**Authors:** Shuai Li, Haibo Si, Shaoyun Zhang, Jiawen Xu, Yuan Liu, Bin Shen

**Affiliations:** Department of Orthopedic Surgery and Orthopedic Research Institute, West China Hospital, Sichuan University, Chengdu, Sichuan Province People’s Republic of China

**Keywords:** Diabetes mellitus, Osteoarthritis, Forgotten joint score-12, Total knee arthroplasty, Enhanced recovery after surgery

## Abstract

**Background:**

Diabetes mellitus (DM) and osteoarthritis (OA) are common diseases that are predicted to increase in prevalence, and DM is a risk factor for OA progression and has a negative impact on the outcome. However, the evidence remains unclear on how it affects patients’ clinical results of total knee arthroplasty (TKA) under enhanced recovery after surgery (ERAS).

**Methods:**

A retrospective single-center study was conducted comparing diabetic and non-diabetic patients who underwent TKA in West China Hospital of Sichuan University between September 2016 to December 2017 under ERAS. Consecutive propensity score matching (PSM) was conducted by 1:1 (DM: non-DM) matching analysis with all baselines as covariates. The primary clinical results were the improvement of knee joint function, the incidence of postoperative complications, and the FJS-12 sensory results 5 years after the operation between DM and Non-DM groups. The secondary clinical results were the postoperative length of stay (LOS), postoperative blood test and total blood loss (TBL).

**Result:**

After PSM, the final analysis included 84 diabetic patients and 84 non-diabetic patients. Diabetic patients were more likely to experience early postoperative complications (21.4% vs. 4.8%, *P* = 0.003), of which wound complications are the most significant (10.7% vs. 1.2%, *P* = 0.022). Diabetic patients experienced longer postoperative LOS with a significant increase in patients with LOS exceeding 3 days (66.7% vs. 50%, *P* = 0.028) and showed less postoperative range of motion (ROM) (106.43 ± 7.88 vs. 109.50 ± 6.33 degrees, *P* = 0. 011). Diabetic patients also reported lower Forgotten joint score (FJS-12) than non-diabetic patients (68.16 + 12.16 vs. 71.57 + 10.75, *P* = 0.020) in the 5-year follow-up and were less likely to achieve a forgotten knee joint (10.7% vs. 1.2%, *P* = 0.022). In additional, Compared with non-diabetics, diabetic patients showed lower hemoglobin (Hb) (*P* < 0.001) and hematocrit (HCT) (*P* < 0.001) and were more likely to suffer from hypertension before TKA (*P* < 0.001).

**Conclusion:**

Diabetic patients show increased risk for postoperative complications, and have lower lower postoperative ROM and lower FJS-12 compared with non-diabetic patients after TKA under ERAS. More perioperative protocols are still needed to be investigated and optimized for diabetic patients.

## Introduction

Total knee arthroplasty (TKA) is one of the most commonly done and cost-effective musculoskeletal surgical procedures with a history of over 40 years, and usage continues to grow worldwide [1]. Projected analyses from different counties all suggest that, even with conservative estimates, the increased use of knee replacement will continue [2]. The success of TKA is based on improving the quality of life for patients with knee arthritis by reducing pain and improving long-term function [1].

About 537 million adults worldwide have diabetes mellitus (DM), and this number is expected to rise annually [3]. The latest studies also show that COVID-19 infection was associated with an increased risk of DM [4, 5]. DM is a risk factor for suboptimal perioperative outcomes in patients undergoing orthopedic surgery [6]. The incidence of infection and cardiovascular events in patients with DM is higher, especially in patients with obesity, which undoubtedly increases the risk of complications during and after TKA [7]. Primary osteoarthritis (OA) is the main indication of TKA. The increase in the prevalence of OA is also the main reason for the rise in the number of TKA [8]. DM and OA are common diseases that are predicted to increase in prevalence [9]. Clinical trials have shown that DM is significantly associated with OA [10]. A previous review and meta-analysis reported that the prevalence of DM was 14.4% in patients with OA, while OA was 29.5% in patients with DM [11]. Increasing evidence from the laboratory supports an adverse effect of DM on the development, severity and therapeutic outcomes for OA [12].

Forgotten joint score (FJS-12) is a 12-item scoring table used to evaluate patients' understanding of artificial joints in various activities of daily life [13]. FJS-12 has been applied to the post-TKA evaluation of different types of people in the past few years [14]. Compared with the traditional WOMAC score, FJS-12 has advantages in evaluating the knee joint sensation of patients [15]. At present, there are only few studies on postoperative FJS-12 assessment of DM patients. The consequence of DM on OA outcomes is a question of research interest. This study compared the clinical and functional characteristics of elderly diabetic and non-diabetic patients after TKA and assessed the effect of DM on outcomes and prognosis of TKA.

The concept of enhanced recovery after surgery (ERAS) concept was first proposed in 1997 [16]. ERAS has been shown to promote early functional recovery after TKA [17]. DM is a risk factor for OA progression and is not conducive to postoperative rehabilitation following TKA [18]. However, research on ERAS for clinical results’ improvement in diabetic patients is still lacking.

## Method

### Study design

This study was approved by our institutional review board of the West China Hospital of Sichuan University (No. 201302007). Patients’ data were extracted from a single institution’s joint arthroplasty registry with prospectively collected data. We identified 1142 adult patients who underwent primary elective TKA between September 2016 to December 2017. In addition, patients with bilateral TKA (*n* = 5), TKA for non-OA reasons (*n* = 100): rheumatoid arthritis (*n* = 91), post-traumatic arthritis (*n* = 7), gouty arthritis (*n* = 1), hemophilic arthritis (*n* = 1), missing information (*n* = 19) data were excluded. Subsequently, the information of 1018 patients of which 137 (12.6%) had DM were collected for this study. Been diagnosed with taking anti-DM drugs, fasting blood glucose > 7.0 mmol/L or HbA1c > 6.5% were classified as diabetic patients, while not taking anti-DM drugs, HbA1c < 6.5% and fasting blood glucose 7.0 mmol/L were classified as non-diabetic patients [7]. In addition, only well-controlled DM (HbA1c < 8.0%) were allowed to proceed with TKA. Poorly controlled DM (HbA1c > 8.0%) were referred to primary care providers or endocrinologists for better glycemic control before consideration for surgery. Perioperatively, glycemic control for DM was optimized using sliding scale insulin and a diabetic diet during inpatient. Participate in the clinical trial voluntarily and sign the informed consent form, with good compliance.

**Fig. 1:**
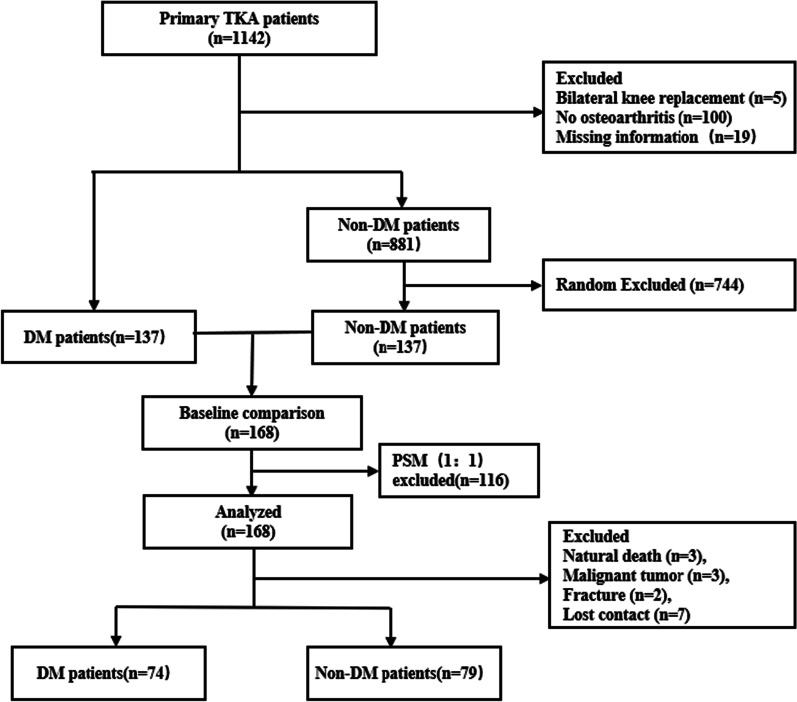
The flowchart of the patient selection process and the patients were divided into two groups. PSM: propensity score matching; TKA: total knee arthroplasty; DM: diabetes Mellitus

Patients were divided into a DM and non-DM group according to whether they had DM before surgery. Their procedures and medical records, which are linked to the unique identifier, were tracked using the Hospital Information System (HIS) at the West China Hospital of Sichuan University. Variables collected include patient demographics (age, gender, body mass index (BMI)), course of the OA, side for TKA, preoperative range of motion (ROM), comorbidities (peripheral artery disease, intramuscular venous thrombosis, hypertension, osteoporosis, stroke, coronary heart disease, arrhythmia, liver diseases, preoperative blood test result: hemoglobin (Hb), hematocrit (HCT), albumin (Alb), international normalized ratio (INR), C-reactive protein (CRP) and patients’ blood volume (PBV). PBV was calculated by the Nadler formula [19]. We selected 137 non-diabetic patients based on the date of surgery and compared them with diabetic patients for preoperative baseline. Then, consecutive propensity score matching (PSM) was conducted by 1:1 (DM: non-DM) matching adjusted all baselines. Finally, 84 diabetic patients and 84 non-diabetic patients were included for statistical analysis as a matched cohort (Fig. 1).

### ERAS protocol

Enhanced recovery after surgery (ERAS) protocol was accepted by all patients [20, 21]. In the preoperative stage, the patient's comorbidities are optimized. For example, once hypertension is diagnosed, the blood pressure is optimized to be lower than 140/90 mmHg. Patients are advised to quit smoking and alcohol before surgery. Professional nurses and physiotherapists educated patients before surgery including perioperative pain management, preoperative physical therapy (such as knee ROM and active quadriceps exercise), postoperative rehabilitation plans, and arrangements needed at home after discharge.

In the operative stage, all surgeries were performed under general anesthesia using the standard mid-vast approach in the intraoperative period. Intravenous tranexamic acid and antibiotics were given during surgery. All patients received ropivacaine for postoperative pain relief. We did not use negative suction drains at the wound site. Urinary catheters were not used in all patients. Intraoperative body temperature was controlled using warm saline infusions.

In the postoperative stage, all knees are kept elevated over the pillow and covered with a cold compress bag. Subcutaneous injection of low molecular weight heparin was taken in all patients to prevent deep venous thrombosis prophylaxis. Intravenous nonsteroidal anti-inflammatory drugs (NSAIDs) are routinely used and oral opioids are added for analgesia if necessary.All patients are recommended to consume adequate high-protein foods after surgery. From the evening following surgery, patients undergo functional training in bed under the guidance of professional nurses and physiotherapists. Hemoglobin and hematocrit levels were determined on the first and third days after surgery. All patients were fully mobile with walking aids the day after surgery. Most patient was discharged from the hospital about 3 days after the surgery. They were prescribed oral NSAIDs and analgesics and instructed to continue their rehabilitation exercises at home.

### Outcome evaluation

Our primary interest was postoperative complications after TKA. We have reviewed the medical records of all patients in His system so that we can collect the occurrence of these complications as comprehensively as possible. In addition, we also review and supplement the situation of these complications in the later follow-up. Postoperative complications mainly include wound complications (hematoma, seroma, exudation, ecchymosis around the wound), pulmonary infection, uncontrollable hyperglycemia, fever, arrhythmia, deep venous thrombosis of lower limb and prosthesis-related complications (periprosthetic fracture, periprosthetic infection, periprosthetic osteolysis, prosthetic loosening and dislocation. In addition, we also evaluated the improvement of knee joint function, mainly including the degree of ROM after surgery, and the FJS-12 in 5 years follow-up. Postoperatively, these outcomes were collected when patients returned to the outpatient clinic for follow-up. For patients who did not return for follow-up, this information was collected over the telephone.

The FJS-12 scale consists of 12 questions. The answers to all questions are never, rarely, sometimes, most, or "not related to me", corresponding to 0 to 4 points and a missing value respectively. The higher the score of the FJS-12 scale, the more likely the patient is to forget that they have TKA. The English version of FJS-12 has been proven reliable and effective and has been translated into multiple languages [22]. All patients were followed up for a minimum of 5 years. The passing threshold of FJS-12 is 40.63, and a score higher than 84.38 can be regarded as realizing a forgotten joint [15].

The secondary outcomes were postoperative LOS, Hb, HCT, anemia (Hb < 120 g/L in women and < 130 g/L in men were recognized as anemia), ALB and total blood loss (TBL). The postoperative LOS was the period from the operative day to discharge. Once admission, all patients were examined for preoperative Hb, HCT and Alb. After surgery, the lowest levels of Hb, HCT and ALB were determined for all patients as the postoperative Hb, HCT and Al. TBL was evaluated by the gross formula [23].

### Statistical analysis

Data description was based on means and standard deviation for continuous variables and absolute and relative frequencies for categorical variables. A standard t-test was used for continuous variables while the Chi-Squared test was applied for categorical variables. Mann–Whitney U test was applied for comparison among diabetic patients and non-diabetic patients who underwent TKA for uneven distribution. Statistical analysis was performed using SPSS 25.0 with statistical significance set to *P* < 0.05.

## Result

One hundred thirty-seven diabetic patients and one hundred thirty-seven non-diabetic patients who underwent primary elective TKA between September 2016 to December 2017 were included for statistical analysis to evaluate the baseline difference between the DM group and non-DM group. After consecutive 1:1 PSM, a matched cohort consisting of 84 diabetics patients and 84 non-diabetic patients without significant baseline differences was established to evaluate clinical outcomes. One hundred fifty-three patients (91.6%) completed their post-operative 5-year follow-up.

### Baseline

Characteristics of the diabetic and non-diabetic patients are shown in Table 1. Before PSM, diabetic patients showed higher ages when they received primary TKA surgery (69.94 ± 7.50 vs. 66.42 ± 8.80 years, *P* < 0.001). In terms of preoperative blood check results, preoperative hemoglobin (127.64 ± 12.78 vs. 133.70 ± 12.70 g/L, *P* < 0. 001) and HCT (39.62 ± 3.71 vs. 40.61 ± 3.30% *P* < 0. 001) of diabetic patients were lower than non-diabetic patients. However, there was no statistical difference in total blood volume between the two groups. The proportion of patients with preoperative CRP in diabetic patients was 29.9%, while that in non-diabetic patients was 19.0%. There was a statistical difference between the two groups (*P* = 0.035). Comorbidities of diabetic and non-diabetic patients can be seen in Table 2. Diabetic patients showed a higher rate of hypertension compared with non-diabetic patients (57.7% vs. 37.2%, *P* < 0.001). However, there was no significant difference between other comorbidities such as osteoporosis, liver disease, heart-related disease, and stroke. After PSM, the resultant diabetic and non-diabetic patient cohorts show no significant difference among preoperative baseline characteristics.

**Table 1:** Demographics and preoperative blood test between DM and Non-DM groups

Variables	before PSM			After PSM		
	DM (*n* = 137)	Non-DM (*n* = 137)	*P* value	DM (*n* = 84)	Non-DM (*n* = 84)	*P* value
Agee (years)	69.94 ± 7.50	66.42 ± 8.80	**0.001**	68.10 ± 7.69	68.15 ± 7.82	0.960
Gender (Female)	114 (83.2%)	103 (75.2%)	0.102	68 (81.0%)	70 (83.3%)	0.687
Course (years)	9.50 (6.25.12.75)	10.00 (5.00,15.00)	0.513	10.00 (7.50,12.50)	10.00 (5.00,15.00)	0.816
BMI (kg/m2)	25.77 (23.50, 28.04)	25.53 (23.20, 27.86)	0.603	25.51 (23.03, 27.99)	25.77 (23.36, 28.11)	0.811
Side for TKA (Right)	77 (56.2%)	71 (51.8%)	0.467	45 (53.6%)	45 (53.6%)	1.000
PBV (ml)	3747.43 ± 492.39	3793.29 ± 540.63	0.459	3757.50 ± 535.38	3734.18 ± 527.94	0.775
Preoperative-ROM	92.86 ± 19.94	94.53 ± 17.07	0.485	93.15 ± 20.30	94.40 ± 17.69	0.671
Preoperative blood test						
HCT (%)	39.62 ± 3.71	0.41 ± 0.03	**0.001**	39.62 ± 3.71	39.60 ± 2.97	0.963
Hb (g/L)	127.64 ± 12.78	133.70 ± 12.70	**0.001**	130.25 ± 13.37	130.02 ± 11.46	0.906
ALB (g/L)	43.93 ± 3.53	43.75 ± 3.03	0.665	43.95 ± 3.73	44.08 ± 2.87	0.796
CRP (> 5 nmol/L)	41 (29.9%)	26 (19%)	**0.035**	25 (29.8%)	21 (25.0%)	0.489
INR	0.98 ± 0.10	0.97 ± 0.07	0.459	0.98 ± 0.12	0.98 ± 0.07	1.000
TC (mmol/L)	39 (28.5%)	42 (30.7%)	0.251	4.73 ± 1.18	4.73 ± 0.97	0.997

Statistically significant *P* values are in bold

*PSM* propensity score matching, *BMI* body mass index, *TBV ROM* range of motion, *HCT* hematocrit, *Hb* hemoglobin, *ALB* albumin, *CRP* C-reactive protein, *INR* international normalized ratio, *DM* diabetes mellitus, *TC* Total cholesterol, *PBV* patients’ blood volume

**Table 2:** Comorbidities between DM and Non-DM groups

Variables	before PSM			After PSM		
	DM (*n* = 137)	Non-DM (*n* = 137)	*P* value	DM (*n* = 84)	Non-DM (*n* = 84)	*P* value
Peripheral artery disease	8 (5.8%)	13 (9.5%)	0.256	4 (4.8%)	4 (4.8%)	1.000
Intramuscular venous Thrombosis	6 (4.4%)	6 (4.4%)	1.000	2 (2.4%)	3 (3.6%)	1.000
Hypertension	79 (57.7%)	51 (37.2%)	**0.001**	42 (50.0%)	39 (46.4%)	0.643
Osteoporosis	35 (25.5%)	28 (22.4%)	0.315	19 (22.6%)	19 (22.6%)	1.000
Stroke	8 (5.8%)	13 (9.5%)	0.256	7 (8.3%)	4 (4.8%)	0.533
Coronary heart disease	9 (6.6%)	7 (5.1%)	0.606	5 (6.0%)	3 (3.6%)	0.717
Arrhythmia	15 (10.9%)	8 (5.8%)	0.127	9 (10.7%)	7 (8.3%)	0.599
Liver disease	17 (12.4%)	19 (13.9%)	0.721	12 (14.3%)	6 (7.1%)	0.134

Statistically significant *P* values are in bold

*PSM* propensity score matching, *DM* diabetes mellitus

### Postoperative blood test

Table 3 describes the postoperative blood test and clinical outcomes between diabetic and non-diabetic patients. There were no significant differences in postoperative HCT, Hb, TBL or anemia rate between diabetic patients and non-diabetic patients.

**Table 3:** Postoperative outcomes between DM and non-DM groups

Variables	DM (*n* = 84)	Non-DM (*n* = 84)	*P* value
*Postoperative blood test*			
HCT (%)	34.68 ± 3.54	34.87 ± 3.57	0.724
Hb (g/L)	114.93 ± 12.98	115.35 ± 12.76	0.834
Anemia	59 (70.2%)	58 (69.0%)	0.867
ALB (g/L)	38.58 ± 3.44	38.46 ± 3.22	0.805
Postoperative ROM	106.43 ± 7.88	109.50 ± 6.33	**0.011**
ROM improvement	13.27 ± 20.92	16.41 ± 17.73	0.648
TBL (ml)	185.69 ± 126.24	175.07 ± 108.13	0.559
Postoperative LOS	4.07 ± 1.57	3.68 ± 0.96	0.052
Postoperative LOS > 3 days	56 (66.7%)	42 (50%)	**0.028**
Postoperative complications	18 (21.4%)	4 (4.8%)	**0.003**
Pulmonary infection	3 (3.6%)	0 (0.0%)	0.244
Uncontrollable Hyperglycemia	5 (6.0%)	0 (0.0%)	0.069
Wound complications	9 (10.7%)	1 (1.2%)	**0.022**
Fever	2 (2.4%)	1 (1.2%)	1.000
Arrhythmia	1 (1.2%)	0 (0.0%)	1.000
Intramuscular venous thrombosis (new)	1 (1.2%)	1 (1.2%)	1.000
Periprosthetic fractures	0 (0%)	1 (1.2%)	1.000

Statistically significant *P* values are in bold

*DM* diabetes mellitus, *ROM* range of motion, *HCT* hematocrit, *Hb* hemoglobin, *ALB* albumin, *TBL* total blood loss, *LOS* length of stay

### Postoperative LOS

Although there was no significant difference in postoperative LOS between diabetic patients and non-diabetic patients (4.07 ± 1.57 vs. 3.68 ± 0.96 days, *P* = 0.052), diabetic patients got a higher rate of postoperative LOS > 3 days (66.7% vs. 50%, *P* = 0.028).

### Postoperative complications

In this study, postoperative complications mainly include pulmonary infection, wound complications, fever, uncontrollable hyperglycemia, arrhythmia, intramuscular venous thrombosis and prosthesis-related complications. The diabetics had an increased overall incidence of postoperative complications compared with the non-diabetics (21.4% vs. 4.8%, *P* = 0.003). In additional, diabetics exhibited significantly higher rates of wound complications (10.7% vs. 1.2%, *P* = 0.022). The rate of uncontrollable hyperglycemia was 6.0% in diabetics versus 0.0% in non-diabetics and was found to be not significantly different (*P* = 0.069). The rates of other complications were not statistically different (Table 3). Pulmonary infection, uncontrollable hyperglycemia and arrhythmia were not observed in the non-diabetics.

### Knee ROM

The mean ROM improvement was 13.27 (SD: 20.92) for diabetics and 16.41 (SD: 17.73) for non-diabetics. There was no significant difference in ROM improvement between diabetics and non-diabetics (*P* = 0.648). However, diabetics showed significantly poorer postoperative ROM than non-diabetics (106.43 ± 7.88 vs. 109.50 ± 6.33 degrees, *P* = 0. 011).

### FJS-12

Because of missing data, FJS-12 at 5 years postoperatively was only available for 153 patients (74 diabetics, 48.9%). Of the 15 missing data, 3 patients died of malignant tumors, 3 patients died of natural death, 2 patients could not be scored due to fracture (1 periprosthetic fracture, 1 non-prosthetic periprosthetic fracture), 7 patients lost contact. The FJS-12 score in diabetic patients at 5 years follow-up was lower than non-diabetics (68.16 + 12.16 vs. 71.57 + 10.75, *P* = 0.020). Non-diabetic patients were more likely to reach forgotten joints than diabetic patients (16.5% vs. 7.4%, *P* = 0.028).

## Discussion

In this study, we compared the effectiveness of DM on the clinical outcomes of patients who underwent TKA. We found that diabetic patients had poorer clinical outcomes compared with non-diabetics, including higher rates of clinical complications, lower postoperative ROM, lower FJS-12 and higher rates of postoperative LOS > 3 days,. This issue is important because the prevalence of DM is increasing and one of the most frequent diseases in individuals with diabetes is OA [24] (Table 4).

**Table 4:** FJS-12 of 5 years follow up between DM and Non-DM groups

Variables	DM (*n* = 74)	Non-DM (*n* = 79)	*P* value
FJS-12	68.16 + 12.16	71.57 + 10.75	**0.020**
Reach forgotten joint	9 (7.4%)	21 (16.5%)	**0.028**
	112 (92.6%)	106 (83.5%)	
Not Passing	4 (3.3%)	1 (0.8%)	0.338
	117 (96.7%)	126 (99.2%)	

Statistically significant *P* values are in bold

*DM* diabetes mellitus, *FJS-12* forgotten joint score-12

Anemia is a common complication of DM and it is often neglected [25]. Our study shows that diabetic patients show lower perioperative Hb and HCT. Insufficient EPO production caused by hyperglycemia may be the main reason for early anemia in diabetic patients [26]. Blood management is an important part of ERAS [16], higher preoperative hemoglobin level in a certain range is related to the reduction of complications after TKA, Cai and his group found that the LOS decreased with increased hemoglobin values when Preoperative Hemoglobin was beyond 140 g/L [27]. Further studies show that preoperative hemoglobin ≥ 140 g/L is associated with a lower risk of postoperative complications in patients undergoing primary TKA [28]. Li and his co-worker found that preoperative anemic patients had higher rates of complications, extended LOS, and mortalities [29], and preoperative anemia also affected the postoperative blood transfusion rate [30]. Our study also shows no significant difference in the TBL, postoperative Hb and HCT after PSM, suggesting that surgeons should pay more attention to the preoperative blood management of diabetic patients [31]. In later studies, we will try to divide diabetic patients into different groups and explore the factors that influence the clinical outcomes of diabetic patients after TKA.

Our result showed that both groups experienced significant improvements in postoperative ROM, but diabetic patients experienced lower postoperative ROM (*P* = 0.011) than non-diabetics. Previous studies shared the same opinion, Osamu Wada and his group found that diabetic patients had significantly lower knee flexion and smaller improvements in the new Knee Society Score than nondiabetics [32]. Further studies show that even at long-term follow-up, postoperative ROM is inferior in diabetics to non-diabetics [33]. Many researchers have tried to explain this phenomenon, and one hypothesis is that intracellular hyperglycemia and advanced glycation endproducts (AGEs) induced cellular damage and AGE-cross-links to collagen extensively accumulate in the skin, tendons and ligaments play an important role in the poor ROM of diabetics [34, 35]. We will go further research to study the effect of blood sugar and HbA1c levels on postoperative ROM following TKA surgery.

Our results have also shown that compared with non-diabetics, diabetics got higher rates of postoperative complications (*P* = 0.003), especially wound complications. (*P* = 0.022) and higher rates of postoperative LOS > 3 days (*P* = 0.028). The higher rates of wound complications in diabetic patients may be related to delayed collagen synthesis and impaired phagocytosis induced by hyperglycemia [36]. Previous results have shown that patients with DM are more prone to wound complications after surgery [37]. Further studies show that patients with diabetes who require insulin or oral hypoglycemic drugs have an increased chance of LOS > 4 days and higher rates of postoperative complications compared with non-diabetic patients, and our findings are consistent with these results. Patients with pharmacologically treated diabetes undergoing TKA show the highest rate of LOS > 4 days, while patients with insulin-treated diabetes show the highest rate of postoperative complications [38]. In addition, many clinical trials also found that hyperglycemia would increase the risk of postoperative infection at the surgical site and increase the rehospitalization rate [39, 40]. And another study showed that the uncontrolled-diabetes group had the greatest TKA readmission and complication odds.

The FJS-12 scale was created in recent years to evaluate the degree of knee joint forgotten, existing studies have found that multiple factors have negative correlations with FJS-12 after surgery, such as preoperative depression, postoperative patellar subluxation [41] and knee instability [42]. Recent studies have found that the FJS-12 score of diabetic patients is lower than that of non-diabetic patients 1 year after surgery, and the difference is more obvious in female patients [43]. To our knowledge, no study has evaluated the FJS-12 score in diabetic patients 5 years after TKA surgery. Our research has filled the gap in this area and found that there is a statistical difference in FJS-12 scores of diabetic patients five years after surgery compared with non-diabetic patients (*P* = 0.024). At the same time, the opportunity to reach forgotten joint (FJS-12 > 84.38) in diabetic patients is different from that of non-diabetic patients (6.8% vs. 24.1%, *P* = 0.003), this has a certain guiding significance for diabetic patients who choose TKA, and we will try to find specific factors influencing postoperative FJS-12 in diabetic people in further studies.

## Limitation

Although our study analyzed post-TKA outcomes of diabetic and non-diabetic patients, it has some limitations. First, this is a single-center retrospective study, which may weaken the generalizability of the results. A multicentre study may guarantee that our results are more convincing. Second, the number of people included in the study is insuffcient to effectively study diseases with low incidence rates such as periprosthetic fractures and infections. Third, We only performed FJS-12 score collection at five years postoperatively, which prevented us from comparing trends in postoperative FJS-12 changes. Finally, we will investigate measures to reduce adverse outcomes after TKA in diabetic patients in future studies.

## Conclusion

By utilizing a pseudo-randomized propensity-matched cohort analysis of diabetic patients versus non-diabetic patients undergoing TKA, we found that diabetic patients at an increased risk for postoperative complications and wound complications, and have lower postoperative ROM and lower FJS-12 compared with non-diabetic patients after TKA under ERAS. Clinical outcomes for TKA may be less desirable in diabetic patients than in non-diabetic patients. Larger multicenter, prospective studies are needed to further confirm this conclusion. Meanwhile, we will conduct further researches and try to find which measure can be taken to lighten the impairment in clinical results of TKA under ERAS caused by DM. Our results urge the development of clinical guidelines for the approach to preoperative diabetes before TKA.

## Data Availability

The datasets used and/or analyzed during the current study are available from the corresponding author on reasonable request.

## Abbreviations

Alb: Albumin
BMI: Body mass index
CRP: C-reactive protein
DM: Diabetes mellitus
ERAS: Enhanced recovery after surgery
FJS-12: Forgotten joint score
HCT: Hematocrit
INR: International normalized ratio
LOS: Length of stay
NSAIDs: Nonsteroidal anti-inflammatory drugs
OA: Osteoarthritis
PBV: Patients’ blood volume
PCA: Patient-controlled analgesia
PONV: Postoperative nausea and vomiting
PSM: Propensity score matching
ROM: Range of motion
TBL: Total blood loss
TKA: Total knee arthroplasty
